# Anæsthesia in Midwifery

**Published:** 1853-03

**Authors:** Henry Miller

**Affiliations:** Professor of Obstetric Medicine in the University of Louisville


### SELECTIONS

FROM

### FOREIGN AND HOME MEDICAL JOURNALS.

---

*Anæsthesia in Midwifery.* By Henry Miller, M. D., Professor of Obstetric Medicine in the University of Louisville.

(From the Transactions of the Kentucky State Medical Society.)

*First. The mode of using Anæsthetics*—Several chemical liquids, of a volatile nature, have been ascertained by experiment to possess anæsthetic properties,—such as sulphuric ether, perchloride of formyle or chloroform, chloride of hydrocarbon, nitrate of ethyle, bisulphuret of carbon, &c.—Agreeing in the common property of producing, more or less perfectly, the anæsthetic state, each of these agents is imbued with properties peculiar to itself, rendering it, more or less, suitable for the particular use in question. After many experiments upon himself and others, Dr. Simpson was induced to give the preference to chloroform over all its congeners. Chloroform is preferable to sulphuric ether,—the only one of the list that can be put in competion with it,—in several respects: it is more portable, a small quantity sufficing to produce the effect; it is less exciting and more agreeable to inhale; and its action is more rapid and complete, and generally more persistent.

Chloroform is the only agent which has been used by the writer, and he has had no reason to be dissatisfied with it, or inducement to try others. In its exhibition, he has never used anything but a lady's handkerchief, folded the size of his hand, and held in its palm, rendered hollow by flexing the fingers. The handkerchief is sprinkled with chloroform, either by pouring or shaking the vial containing it while the handkerchief is held on its mouth. Perhaps a drachm may be required for its first wetting, and it is then held, in the hollow of the hand, over the nose and mouth, in pretty close approximation. The patient must be encouraged to inhale it until she is quieted, or until slight snoring indicates that she has fallen into a state resembling natural sleep, when the handkerchief must be removed and folded in the hand, to prevent, as much as possible, the evaporation of the chloroform. During or immediately previous to each returning pain, the handkerchief is to be held to the nose and mouth; and must be sprinkled afresh with the chloroform, from time to time. When distention of the perineum gives warning of the advent of the child, to relieve the more excrutiating pains which are then endured, the patient should be made to enhale more freely until a greater degree of insensibility is induced than had existed previously. Until this period it is not essential that the inhalations be pushed to the extent of producing complete insensibility. It may be, and frequently is used by the writer as a *benumber* of pains at first, and is more liberally exhibited toward the conclusion, so as to annul the anguish and avert the shock which would otherwise be endured by the patient. The accoucheur need not, and indeed cannot himself administer the chloroform throughout the labor without neglecting his indispensable duties, especially toward its termination. The writer's practice is, having commenced the administration and observed its effects, to commit the handkerchief and chloroform vial to any female friend, who may chance to be present, and direct by signals its further use.

*Secondly—The Benefits of Anæsthetics. First: In Ordinary Labor, as an Anodyne.* Whatever doubts may have existed formerly, the experience of our own times has demonstrated that pain is not an essential component of parturition in the human species, any more than in the inferior animals. There are, truly, mechanical impediments peculiar to woman, incident to her erect position, and imposed as a tribute for her superior rank in the scale of being. But to surmount these, she is benevolently provided with a muscular apparatus, superior in point of strength to what belongs to any of the inferior creatures; and, in obeying the requisition made upon it, when the fœtus is to be expelled this apparatus may be exerted, to the utmost of its capabilities, without the accompaniment of pain. There is no more curious phenomenon (curious, because we are so accustomed to witness suffering in travail,) than that which we now often see in the lying-in chamber, a woman in the strong throes of child birth, straining every auxiliary muscle in aid of the powerful contracting uterus, while the breath is held and the big drops of sweat stand on her brow, and yet she feels no pain, even though consciousness may remain. The motor nervous power is unimpaired while the sensory is temporarily paralyzed.

If, then, pain be not essential, we are strongly tempted to suspect that it is *unnatural*, and when we consider that among the many families of our race, there is almost a complete exemption from it, our suspicion is converted into assurance. When it may be truly predicated of any physiological function, that it is unnaturally performed, it must be concluded that the organs concerned in it are in a morbid condition, and this was a doctrine held and promulgated by Dr. Rush, which anæsthesia has contributed not a little to confirm. "The philosophers," wrote Dr. Rush, "in describing the humble origin of man, say that he is formed '*inter stercus et urinam.*' The divines say that he is 'conceived in sin, and shapen in iniquity.' I believe it to be equally true, and alike humiliating, that he is conceived and brought forth in disease." This disease, according to the wise and benevolent author, appears in pregnancy and parturition. In pregnancy there is inflammation of the uterus, evinced by all the usual phenomena of that morbid state, in other parts of the body, such as swelling and enlargement, hemorrhage, a full, quick and tense or frequent pulse, sizy blood, and the formation of a membrane upon the internal surface of the uterus, similar to that which is formed upon other inflamed mucous surfaces. "Parturition," he continues; "is a higher grade of disease than that which takes place in pregnancy," consisting of "convulsive or clonic spasms in the uterus, supervening its inflammation, and accompanied with chills, heat, thirst, a quick full, tense, or a frequent and depressed pulse, and great pain."

For this disease of pregnancy and parturition, the great remedy of Dr. Rush was blood-letting, and perhaps his partiality for the lancet gave too much depth and intensity to the coloring of the picture which he has drawn of the pregnant and parturient states. After all due abatement, however, it must be allowed, by the candid inquirer, that in the actual condition of civilized society, much more suffering and danger await women, at such junctures, than in other conditions more simple and natural. We are not going to sing the praises of barbarism,—for we do not believe that mankind were intended by nature to live by the chase, and sleep in wigwams,—but it cannot be doubted that the habits of females, in highly civilized countries,—their luxurious living, their modes of dress, their neglect of exercise, and erroneous mental and moral culture,—tend to unduly exalt the nervous system, while the muscular is depressed and enervated. Hence, with not a few of them, pregnancy is but an interminable train of anomolous maladies, and parturition an enfeebled struggle, in which sensibility displays its usurped ascendancy over muscular contraction.

To allay this morbid sensibility,—to assuage the pain and anguish which are its product,—is surely a task that the obstetrician cannot deem unworthy of his best exertions. If it be esteemed, upon the authority of Lord Bacon, "the office of a physician, not only to restore health, but to mitigate pain and dolors," he will find no fairer field for the exercise of this enviable prerogative, for there are no "dolors" that are at all comparable to those of morbid parturition; and, in the qualified sense which has been explained, nearly every case with which we are concerned is of this nature.

But the benefits resulting from anæsthetics in Midwifery are not confined to the alleviation or annulment of pain. There is nothing established by more indubitable evidence than the greater freedom from the aches and ills of childbed, enjoyed by those who have been subjected to their influence. They complain of little or no weariness or soreness, and make better and more rapid recoveries. The writer was early struck, in a remarkable manner, with this fact, as he thinks every observant practitioner must be who uses chloroform, before his attention was particularly drawn to the testimony of others. This testimony it is needless to quote in detail ; it may be found, in great abundance, in nearly all the narratives of cases, and in all the communications that have been written on the subject. What adds greatly to its credibility is the further fact, that in most instances, it is the spontaneous declaration of the patients,—their unprompted and grateful tribute,—who, if they have borne children before, under the old régime of strong cries and groans, wonder to find themselves so much more comfortable—so much more like themselves in their best estate.

*Secondly : The benefits of Anæsthetics in extraordinary Labor.* If it be thus safe and salutary to administer Chloroform in ordinary labor, we should expect still greater benefits from its use in extraordinary labor, involving, as it does, increased suffering and danger. The pain attendant upon natural parturition is perhaps as great as that which is endured in most surgical operations, while there can be no doubt that in some cases of preternatural labor the agony is both greater and more protracted than in any of the operations of Surgery. We refer more especially to delivery by turning in shoulder presentations, when the uterus is emptied of its waters and is molded to the fœtus, —leaving but little space for the hand, and disputing every inch of ground, by the indomitable contraction of its fibres. In such a condition, the patient is not only exposed to excruciating pain, but to the risk of laceration of the womb, or destruction of the child. The imprisonment of the placenta by the hour glass contraction offers another formidable difficulty, the surmounting of which subjects the woman to cruel torture and no slight danger. We see not how a humane practitioner can justify himself in withholding chloroform in such cases, even if it were not otherwise beneficial than as an anyodyne and oblivious antidote of the pain which his ministration must otherwise inflict. The appeal is, however, not to his sympathy alone. There are valid grounds for the belief, that chloroform, in such cases, is not only a pain-annulling but a life-saving medicament. Dr. Channing has collected the statistics of 51 cases of instrumental, preternatural and complicated labor, treated with ether or chloroform, among which were nine cases of presentation of the shoulder,—all the mothers recovered, and six of the children were born alive,—which is more favorable, we think, than the general results of such cases. There were 24 cases of instrumental delivery, viz:—20 forceps and 4 craniotomy; all the mothers recovered, and 15 of the children were born alive, which is also greater success, we think, than generally attends the employment of instruments in Obstetric Surgery.

We do not allege that the statistics of Dr. Channing prove absolutely that chloroform has diminished the mortality of obstetric operations; but they serve, at least, to strengthen the faith, which reasoning is calculated to beget. What is there in manual and instrumental delivery, that enhances its danger more than the additional pain and shock which must be endured? And to neutralize this pain, to foil this shock, must increase the chances of recovery, by counteracting one of the elements of destruction. Pain is depressing in its influence upon the vital energies, and in its greatest intensity may even extinguish life.

We do not doubt that when the subject shall be fairly investigated by treating a sufficiently large number of difficult cases in Midwifery, similar in kind, and circumstances, with and without etherization, a large balance of maternal and fœtal life will be found in favor of etherization. This prediction is justified by the statiscal evidence collected by Dr. Simpson, which proves undeniably that the mortality of one of the most formidable operations in Surgery, viz: amputation of the thigh, has been remarkably diminished by etherization. He gives, in tabular form, the results of this operation in severa extensive Hospitals, from which it appears that the lowest mortality was 36 per cent., or 46 deaths in a 127 cases, in the Glasgow Hospital, wherein ether was not employed; while the same operation upon 145 patients, in an etherized state, but in other respects, under similar circumstances, was fatal in only 37 cases, or 25 per cent. —being a saving of 11 lives in every 100 amputations! This furnishes a strong argument from analogy in favor of the saving efficacy of etherization in Midwifery operations; for between these and surgical there is no difference.

We may advance a step further, and venture to predict that chloroform, administered in natural parturition, may sensibly curtail it of the mortality incident to it independently of any operative procedures on the part of the accoucheur; so that of an equal number of cases of natural labor, treated with and without chloform, fewer of the former will die in childbirth than of the latter. The more fortunate issue of the etherized cases will be mainly attributable to the annulment of pain, which is a pernicious ingredient in the process of parturition; but we would not perhaps greatly err if we were to consider every parturition to be a natural surgical operation, and, therefore, as likely to be benefited by chloroform as the surgical operations of man's device. In the natural operation the dislodgment of the fœtus is effected by the enormous and forced dilatation of the os uteri, vagina and vulva, and the application of a powerful *vis a tergo*, instead of a tractor, which an accoucheur would employ. After the operation is over, the uterus is in a state, which has been compared by Cruveilhier, (*Anat. Path. du. corps. hum.*, *livr.* XIII,) to a wound or the stump of a limb after amputation. Where the placenta and membranes had been attached, the mucous membrane is no longer found, or is only seen in the cervix, and about the orifices of the tubes; every where else, the muscular tissue appears naked. Upon that portion of the uterine surface, which corresponded to the placenta, may be seen large venous orifices, plugged by coagula, resembling the orifices of the veins of the stump after amputation,—the simile is Cruveilhier's, not ours. Here, then, is an extensive solution of continuity, which needs to be repaired, and the process instituted by nature for that purpose, is accompanied by more or less fever, having many points of resemblance to that which follows other wounds, and hence denominated by Cruveilhier traumatic fever.

